# Postulated Vasoactive Neuropeptide Autoimmunity in Fatigue-Related Conditions: A Brief Review and Hypothesis

**DOI:** 10.1080/17402520600568252

**Published:** 2006-03

**Authors:** Donald R. Staines

**Affiliations:** Gold Coast Public Health Unit. Qld. Australia

## Abstract

Disorders such as chronic fatigue syndrome (CFS) and gulf war syndrome (GWS) are characterised by prolonged fatigue and a range of debilitating symptoms of pain, intellectual and emotional impairment, chemical sensitivities and immunological dysfunction. Sudden infant death syndrome (SIDS) surprisingly may have certain features in common with these conditions. Post-infection sequelae may be possible contributing factors although ongoing infection is unproven. Immunological aberration may prove to be associated with certain vasoactive neuropeptides (VN) in the context of molecular mimicry, inappropriate immunological memory and autoimmunity.

Adenylate cyclase-activating VNs including pituitary adenylate cyclase-activating polypeptide (PACAP), vasoactive intestinal peptide (VIP) and calcitonin gene-related peptide (CGRP) act as hormones, neurotransmitters, neuroregulators, immune modulators and neurotrophic substances. They and their receptors are potentially immunogenic. VNs are widely distributed in the body particularly in the central and peripheral nervous systems and have been identified in the gut, adrenal gland, blood cells, reproductive system, lung, heart and other tissues. They have a vital role in maintaining cardio-respiratory function, thermoregulation, memory, concentration and executive functions such as emotional responses including social cues and appropriate behaviour. They are co-transmitters for a number of neurotransmitters including acetylcholine and gaseous transmitters, are potent immune regulators with primarily anti-inflammatory activity, and have a significant role in protection of the nervous system against toxic assault as well as being important in the maintenance of homeostasis.

This paper describes a biologically plausible mechanism for the development of certain fatigue-related syndromes based on loss of immunological tolerance to these VNs or their receptors following infection, other events or de novo resulting in significant pathophysiology possibly mediated via CpG fragments and heat shock (stress) proteins. These conditions extend the public health context of autoimmunity and VN dysregulation and have implications for military medicine where radiological, biological and chemical agents may have a role in pathogenesis. Possible treatment and prevention options are considered.


*Clinical & Developmental Immunology*, March 2006; 13(1): 25–39

Taylor & Francis

Taylor & Francis Group

### Postulated vasoactive neuropeptide autoimmunity in fatigue-related conditions: A brief review and hypothesis

DONALD R. STAINES

*Gold Coast Public Health Unit, 10-12 Young Street, Southport, Qld 4215, Australia*

**Abstract**

Disorders such as chronic fatigue syndrome (CFS) and gulf war syndrome (GWS) are characterised by prolonged fatigue and a range of debilitating symptoms of pain, intellectual and emotional impairment, chemical sensitivities and immunological dysfunction. Sudden infant death syndrome (SIDS) surprisingly may have certain features in common with these conditions. Post-infection sequelae may be possible contributing factors although ongoing infection is unproven. Immunological aberration may prove to be associated with certain vasoactive neuropeptides (VN) in the context of molecular mimicry, inappropriate immunological memory and autoimmunity.

Adenylate cyclase-activating VNs including pituitary adenylate cyclase-activating polypeptide (PACAP), vasoactive intestinal peptide (VIP) and calcitonin gene-related peptide (CGRP) act as hormones, neurotransmitters, neuroregulators, immune modulators and neurotrophic substances. They and their receptors are potentially immunogenic. VNs are widely distributed in the body particularly in the central and peripheral nervous systems and have been identified in the gut, adrenal gland, blood cells, reproductive system, lung, heart and other tissues. They have a vital role in maintaining cardio-respiratory function, thermoregulation, memory, concentration and executive functions such as emotional responses including social cues and appropriate behaviour. They are co-transmitters for a number of neurotransmitters including acetylcholine and gaseous transmitters, are potent immune regulators with primarily anti-inflammatory activity, and have a significant role in protection of the nervous system against toxic assault as well as being important in the maintenance of homeostasis.

This paper describes a biologically plausible mechanism for the development of certain fatigue-related syndromes based on loss of immunological tolerance to these VNs or their receptors following infection, other events or de novo resulting in significant pathophysiology possibly mediated via CpG fragments and heat shock (stress) proteins. These conditions extend the public health context of autoimmunity and VN dysregulation and have implications for military medicine where radiological, biological and chemical agents may have a role in pathogenesis. Possible treatment and prevention options are considered.

**Keywords:** *Adenylate Cyclase, autoimmunity, chronic fatigue syndrome (CFS), gulf war syndrome, sudden infant death syndrome (SIDS), vasoactive neuropeptides*

### Introduction

Endogenous adenylate cyclase (AC)-activating vasoactive neuropeptides (VNs) may be implicated in causing some fatigue-related conditions currently having no established explanation such as chronic fatigue syndrome (CFS), Gulf War syndrome (GWS), fibromyalgia and even sudden infant death syndrome (SIDS) (Staines 2004a,b,c). This family of VNs includes pituitary adenylate cyclase-activating polypeptide (PACAP), vasoactive intestinal peptide (VIP) and calcitonin gene-related peptide (CGRP). The possible role of endogenous VN autoimmunity in these conditions is a novel but unproven concept.

This paper reviews evidence for these conditions resulting from possible immune dysfunction or other pathologies associated with VNs or their receptors. Causes of these aberrations may include a range of insults such as infection, chemical and biological agents, radiological and physical and psychological stressors including trauma. The hypothesis is explored that infections, possibly exhibiting molecular mimicry with these VNs, or alternatively VN responses from any cause, may provoke adverse autoimmune sequelae

Correspondence: D. R. Staines, Gold Coast Public Health Unit, 10-12 Young Street, Southport, Qld 4215, Australia. Tel: 61 7 55097202/61 414 278 031. Fax: 61 7 5561 1851. E-mail: don_staines@health.qld.gov.au

ISSN 1740-2522 print/ISSN 1740-2530 online © 2006 Taylor & Francis
DOI: 10.1080/17402520600568252

affecting VNs or their receptors and/or their gene expression. While some of these conditions may resolve over time, others may have catastrophic (e.g. SIDS) or long-term sequelae. Variations in outcomes may relate to the degree of long-term perverse immunological events.

This paper also proposes that autoimmune dysfunction of these VNs may represent a family of disorders mediated by significant pathophysiology such as neuronal apoptosis via dysregulation of key biochemical and epigenetic pathways which may be mediated in part by CpG DNA fragments and heat shock (stress) proteins.

### Vasoactive neuropeptide roles and functions

AC-activating VNs have significant commonality between species and are strongly preserved in evolutionary terms indicating their crucial roles for survival. Substantial amino acid sequence homology exists between them, suggesting evolution from a common ancestral gene, and they demonstrate some degree of overlap of structure and function as well as potential for immunological cross-reactivity. These VNs belong to the secretin-glucagon super-family, exerting significant control over carbohydrate and lipid metabolism. They have important roles in vasodilation, neurotrophism, nociception, neuroregulation and neurotransmission including thermoregulation, cardio-respiratory control, balance and vestibular function, emotional and intellectual functioning including memory and concentration, and immunological and hormonal modulation.

They exert their effects at high level in controlling central and peripheral nervous systems and hypothalamic–pituitary–adrenal axis functions. Other vital functions regulated in the brain include olfaction, feeding and reproductive behaviours, circadian rhythm, and sleep–wake cycles. They and their receptors are expressed at important peripheral sites such as heart, gut, blood, lung, pancreas, liver and urogenital systems (Ishizuka et al. 1992, Arimura 1998, Sherwood et al. 2000, Hannibal 2002, Hashimoto 2002, Ganea et al. 2003). Compromise of their function is likely to have serious consequences for homeostasis.

Endogenous opioid activity is functionally related to cytokine and VN activity suggesting that pain mediation and perception may be altered in conditions where endogenous opioid function is impaired through VN mechanisms (Wilderman and Armstead 1997, Peterson et al. 1998). Nitric oxide (NO) metabolism is implicated in immunomodulation as well as possibly mediating chemical sensitivity in these conditions suggesting a plausible mechanism for some concurrent symptoms in CFS and GWS (Pall 2002, Onoue et al. 2002). Synaptic plasticity and pain behaviours are critically mediated by CGRP in the amygdala (Han et al. 2005).

VNs influence receptor accumulation and activity for acetylcholine (Fahrenkrug and Hannibal 2004) and other neurotransmitters including carbon monoxide (CO) (Watkins et al. 2004) and NO (Parsons et al. 2005). PACAP is co-located with vesicular acetylcholine transporter in nerve terminals in all mouse adrenomedullary cholinergic synapses (Hamelink et al. 2002) and CGRP is known to induce acetylcholine receptor expression in rat soleus muscle (Buffelli et al. 2001). There is a known association of VPAC2 receptors with acetylcholine and muscle function (Hinkle et al. 2005).

A number of neurotransmitters are influenced by VNs and include the adrenergic, noradrenergic, cholinergic, histaminergic, GABA-ergic, glutamatergic, dopaminergic and serotonergic systems (Sandvik et al. 2001, Shintani et al. 2003). VIP and PACAP receptors have been demonstrated in guinea pig cerebral cortex and have substantial effects on cyclic adenosine monophosphate (cAMP) production (Zawilska et al. 2005) and PACAP is known to have a potentiating additive effect with adrenaline and noradrenaline on cAMP production in rat cerebral cortex indicating a crucial role in neuroregulation (Nowak and Kuba 2002).

Much central nervous system processing is under VN control to a greater or lesser extent and co-transmitter functions may be a linkage to fatigue mediation and a range of other symptoms in these syndromes. In addition to those functions listed above, high level CNS processes may become compromised in VN disorders including executive functions such as ensuring appropriate behaviours and response to social cues, irritability, emotional lability, planning for the future, empathy and relationship building, verbal skills in problem solving and so on. This would suggest involvement of neuronal connecting pathways between basal ganglia, limbic system of hippocampus and amygdala and pre-frontal and frontal cortices as these pathways are sensitive to compromise (Arnsten and Li 2005).

VNs are mediated by G protein-coupled receptors (GPCR). The secondary transmitter cAMP is generated from adenosine triphosphate (ATP) via AC which is known to exist in multiple isoforms and exhibits a range of processing functions (Nowak and Zawilska 1999), therefore, defects of AC activation will result in impaired cAMP production. VNs operate via multiple signaling processes (Zhou et al. 2002) and have complex interactions with a wide range of neurotransmitters including cross-talk feedback and control mechanisms. Hence, treatment for postulated fatigue-related conditions might include upstream reactivation of cAMP or downstream inhibition of cAMP breakdown (see below).

SIDS may be the manifestation of an acutely acquired autoimmune response to these VNs. Recent minor infection, breathing position and the presence of xenobiotic substances including cigarette smoke may all contribute to loss of immunological tolerance to these VNs or their receptors. This would have potentially catastrophic consequences for cardio-respiratory centres in the brain stem as well as the heart. PACAP, for example, is a powerful respiratory stimulant (Runcie et al. 1995) suggesting vulnerability to respiratory compromise. PACAP-deficient mice suffer sudden neonatal death attributed to respiratory control defects, raising the possibility of VN dysfunction as a potential cause of SIDS (Cummings et al. 2004). Early recognition of potential vulnerability to SIDS, for example, will be of special interest and may contribute to the development of preventive strategies.

Endogenous VNs exert a wide spectrum of immunological functions and have a critical role in homeostasis of neuronal and immune systems through a number of pathways including inhibition of chemokine production in activated microglia. Disturbances in their function are recognised as potential causes of autoimmune disease and they appear to have a role in protecting bystander lysis, a process in the pathogenesis of several autoimmune and inflammatory diseases (Delgado et al. 2002). VNs have a role in maintaining peripheral tolerance by generating tolerogenic dendritic cells (Delgado et al. 2005).

VIP is known to prevent experimental autoimmune uveoretinitis (Keino et al. 2004). PACAP is known to ameliorate experimental autoimmune encephalomyelitis (Kato et al. 2004) and hypoxia (Suk et al. 2004) demonstrating its neuro-protective role. In animal models of EAE, sympathetic nervous system regulation mediated through Th1 cells is significantly promoted by certain other neurotransmitters, e.g. neuropeptide Y (NPY) (Bedoui et al. 2003).

PACAP and VIP exert an extraordinary array of functions in the brain and other organs and peripheral tissues. VIP has been identified in all regions of the brain including cerebral vessels (Fahrenkrug et al. 2000) indicating susceptibility to vasodilatory dysfunction. Neurovascular coupling in vasoactive pathways is mediated via GABA (Cauli et al. 2004). Balance and vestibular functions are regulated by VNs, e.g. CGRP (Kong et al. 2002) and this may have a vascular component (Lyon and Payman 2000). VIP is produced and released by intrinsic neurons in the heart and improves cardiac perfusion and function (Dvorakova et al. 2005). Hence, the vasodilatory role of VNs will be an important function lost through VN failure and may explain a significant extent of the pathophysiology in these conditions by giving rise to hypoperfusion and ischemia.

The role of VNs in protecting the brain from apoptosis is well documented (Falluel-Morel et al. 2004). Apoptosis is known to be higher in SIDS cases than controls with hippocampus and brainstem, including dorsal nuclei, being affected (Waters et al. 1999) and apoptotic neurodegeneration is postulated as the specific pathophysiological mechanism in SIDS (Sparks and Hunsaker 2002).

Mechanisms associated with apoptosis would, therefore, indicate a suitable area for investigation, particularly those mechanisms usually protective against apoptosis. These roles are fulfilled by VNs, for example, ischaemia induced apoptosis in the rat hippocampus is protected by PACAP through inhibition of the JNK/SAPK and p38 signalling pathways (Dohi et al. 2002) and PKA and phosphatidylinositol 3′-OH kinase pathways demonstrate neuroprotective roles in cerebellar granule neurons (Bhave and Hoffman 2004). PACAP is also known to have a neuroprotective effect against a range of insults. Ethanol-induced apoptosis occurs via caspase pathways resulting in DNA fragmentation, mitochondrial permeability and cell death. PACAP, acting via its receptor PAC1, protects against ethanol-induced cell death and may have a therapeutic role in conditions such as fetal alcohol syndrome (Vaudry et al. 2002).

Late-gestation blockade of VIP activity in pregnant mice has shown distinct morphological abnormalities in the somatosensory cortex of offspring and their response to hypoxia being subsequently impaired. A significant arousal deficit was seen in anti-VIP mice, which was not associated with deranged peripheral or brainstem-mediated responses to hypoxia during sleep (Cohen et al. 2002). Compromise of VN function, therefore, has likely major respiratory consequences. This finding may have significant implications for infection in mothers of SIDS victims prior to birth.

Cardiovascular function is in part regulated by VNs. Some variation in the cardiac roles of PACAP exists and is dose dependent, for example, PACAP may promote both tachycardia and bradycardia (Chang et al. 2005). PACAP activates intracardiac postganglionic parasympathetic nerves by shortening the effective refractory period, and has a greater profibrillatory effect than vagal stimulation (Hirose et al. 2001). Neuroexcitability in intracardiac neurons depends on PAC1 receptor activation (DeHaven and Cuevas 2004). Conceivably PACAP opposition, for example, through autoimmune effects on the PAC1 receptor might, therefore, result in decreased cardiac responsiveness.

Brown adipose tissue metabolism and thermal stress have been linked to SIDS. Neonatal adipose tissue is a primary site of cytokine and cytokine receptor action (Gray et al. 2002) and metabolism is a noradrenaline-cAMP-proton pump system. While the precise mechanism of brown adipose tissue metabolism dysfunction is unclear, a combination of factors including metabolic stress, infection, necrosis and vascular hypoperfusion has been suggested (Stephenson and Variend 1987). Hence, VN failure may be mediated through these pathways in SIDS.

### Postulated immunoregulatory dysfunction of vasoactive neuropeptides

The development of autoantibodies to PACAP and VIP in mammalian tissues suggests that immunological tolerance may be readily broken. It has been postulated that depletion of VIP by specific antibodies in autoimmune disease may interfere with VIP regulation of T cells and inflammatory cells and result in further amplification of autoreactive immunological responses (Bangale et al. 2002). Also, as VIP receptors are expressed on T-lymphocytes, VIP could directly affect cytokine production and proliferation of T-lymphocytes (Johnson et al. 1996).

Some researchers have found aberrations of the oligoadenylate synthetase pathway, a key ATP-associated RNase L-mediated antiviral mechanism, in CFS (Nijs and De Meirleir 2005). The oligoadenylate system is considered a major regulator of cell metabolism including cell growth and differentiation, oncogenic stability and apoptosis in addition to antiviral functions (Mykhailyk et al. 2003). This pathway is influenced by VIP (Chelbi-Alix et al. 1991) and cAMP (Itkes 1994) suggesting possible dysregulation with VN compromise.

Autoimmune dysfunction of VNs or their receptors might arise via a number of pathways. Cytosine-guanosine dinucleotide DNA (CpG) fragments are immuno-stimulating or suppressing sequences which may exist in promoter regions of VN receptors and be vulnerable to assault mechanisms such as hypomethylation, resulting in dysregulation of transcriptional/translational capacity. (Pei and Melmed 1995, Lutz et al. 1999). CpG mechanisms may also control secretin gene expression through methylation mechanisms (Lee et al. 2004, Pang et al. 2004) and methylation of gene promoters may have a role in calcitonin/alpha CGRP gene regulation (Broad et al. 1989). The PACAP receptor gene is known to be G + C rich (Aino et al. 1995).

Antibody responses to VNs or VN receptors might hypothetically occur, giving rise to IgM or IgG antibody types, resulting in short or long term autoimmunity to these vital substances. Such mechanisms might also result from mimicry with bacterial residues, giving rise to mistaken recognition of self VN-CpG for bacterial CpG and perverse VN autoimmunity. CpG elements are known to be potent stimulators of immune responses (Krieg 2003).

Heat shock proteins (HSPs) or stress proteins may also play a role in VN autoimmunity or dysfunction. These are important chaperone molecules for proper intracellular functioning of VNs and are known to have a key role in immunoregulation (Srivistava 2002). Moreover, HSPs and CpG are mutually engaged in CpG signalling and recognition (Bandholtz et al. 2003) suggesting that dysfunction or compromise of one may result in compromise of the other, with resulting compromise of VN function (Staines 2005d,e).

High level and intricate homeostasis maintenance appear to be hallmark functions of VNs. As noted above they and their receptors are thought to be immunogenic and are implicated in a range of inflammatory and autoimmune conditions. They also have complex self-regulatory mechanisms involving autoantibody catalysis. The pleiotropic phenotypic presentation of postulated VN autoimmune disorders may reflect the multiplicity of pathogeneses resulting from ligand and receptor diversity.

VN receptors are predominantly G-protein coupled receptors (GPCR) being heterotrimeric seven transmembrane helical structures which control intracellular functioning mostly via cAMP through a number of kinases and related pathways (Vaudry et al. 2000). They and their receptors are widely and commonly distributed through all species and function in remarkably similar ways. As also noted above they exert stimulatory and inhibitory influence on AC, a vital step in cyclic AMP metabolism.

Functional redundancy of these substances and their receptors is limited. Despite high evolutionary preservation of VNs and a high degree of commonality in peptide structure and amino acid sequencing there are only three known receptor types for PACAP and VIP namely PAC1, VPAC1 and VPAC2. It is proposed that this relative lack of functional redundancy makes them vulnerable and susceptible to compromise from different causes resulting in diverse clinical conditions. Autoimmune dysfunction of GPCRs is known but not widely documented. However, some studies have shed some light on autoimmune and toxic processes affecting GPCRs. Also molecular mimicry effects have been documented between some toxins, venoms and infections and VN receptors and are probably due to cross-reactive epitopes (Holz and Habener 1998, Goetzl et al. 2004).

Failure of constitutive or expressed activity of GPCRs may be at the heart of VN autoimmune disorders. The multi-functioning nature of GPCRs and consequent cAMP activation pathways suggest vulnerability to compromised function. These receptors are capable of exhibiting agonism, antagonism and inverse agonism having both stimulatory and inhibitory roles and acting through complex mechanisms (Milligan 2003). Mutations in receptor structure modify their function (Cao et al. 2000). VN autoimmune disorders may prove to operate by aberrant GPCR signaling via these mechanisms (Staines 2005f).

Autoantibodies are found to nuclear and stress proteins (Ro52 and Grp78) in Sjogren's syndrome (SS) (Gordon et al. 2001) and this association is postulated to induce an autoimmune cascade in other conditions (Purcell et al. 2003). Structural similarities with some neuropeptides may suggest their involvement in neurological autoimmune responses. Human muscarinic acetylcholine receptor (mAChR) antibodies from SS sera increase cerebral NO synthase activity and NO synthase mRNA levels in rat frontal cortex indicating central parasympathetic functional deregulation (Reina et al. 2004).

Antibodies to mAChR have been identified in CFS patients (Tanaka et al. 2003a) and autoimmunity to neurotransmitter receptors is also implicated in some psychiatric disorders (Tanaka et al. 2003b) indicating that receptor autoimmunity may result in both primary and secondary psychiatric disorders consequent to receptor pathology. These examples illustrate the multi-faceted and complex roles of VNs and their receptors in neuro-physiology, neuropsychology and autoimmunity.

Genomic expression of null receptor types may play a role in VN autoimmune disorders. Receptors of the "hip" variety are believed to be null variant or fail to transduce signals despite normal ligand binding properties (Zhou et al. 2002). Selective over-expression of these receptors due to defects in genomic expression control may effectively reduce opportunities for proper signal transduction. Alternatively, blocking of otherwise normally transducing receptors by pathogenic autoantibodies may simply block signal transduction perhaps analogously to myasthenia gravis (MG). As these antibodies may be polyclonal with varying degrees of blocking capacity, this may explain heterogeneity in phenotypic expression of VN auto-immune fatigue disorders. In other words fatigue disorders of varying degrees of severity and duration may result.

CpG DNA methylation disorders are being considered as possible causes of neuronal pathology (Iliev et al. 2004). These fragment sequences in mammals are usually methylated and lower in frequency compared with bacterial DNA which are hypomethylated and higher in frequency. In evolutionary terms this represents a "friend or foe" detection system (Goldberg et al. 2000). However, evolutionary residues in mammals have regions reflecting more bacterial characteristics and which are prone to hypomethylation and may predispose to autoimmune phenomena.

Disruption of usual DNA translation mechanisms or epigenetic phenomena associated with these CpG DNA fragments are possible innate sources of auotoimmune reactivity as well as other immunomodulatory responses. VN receptor autoimmunity might conceivably result from perverse immunological memory induced via HSPs or cytosine-guanine (CpG) DNA dinucleotide fragments if directed against the VNs or their receptors. As relative over-expression and under-expression of genomic VN or receptor characteristics may be indicated both immunostimulatory and immunosuppressive CpG fragments may have applications in treatment and prevention and are considered further below.

An intriguing alternative hypothesis is that involving VN receptor desensitisation. Several studies have shown that PAC1 and VPAC receptors undergo homologous desensitisation when pre-exposed to their respective ligands. This mechanism is mediated in retinoblastoma cells via GPCR kinase3 and PKC but not PKA mechanisms (Dautzenberg and Hauger 2001). As noted above, VNs are known to be self-regulated by respective antibodies and catalytic mechanisms. VN fatigue-related disorders may result from loss of control over this neurotransmitter-regulating desensitising mechanism, as it is usually a reversible physiological process. But it is also one subject to impairment depending on other impacting factors such as xenobiotics, infection, immune dysregulation and so on.

Consequences of these anti-receptor mechanisms would be potentially serious as there would be uncoupling of the activity-dependence mechanism which routinely provides potentiation of the AC second messenger system. Pre-synaptic facilitation may be impaired resulting in significant enhancement or exacerbation of the desensitising mechanism. Uncoupling of activity dependency might then occur. Thus, the role of AC as a "coincidence detector" to engage G receptors may be lost. Retrograde signaling may also then be impaired to the pre-synaptic cell resulting in false messages being relayed and VN release excessively prolonged. Interestingly "class-switching" between G stimulatory and G inhibitory receptors occurs (Halls et al. 2005) and this may also explain GPCR dysfunction.

Loss of this functionality may also result in loss of associativity which normally enhances the efficiency of this messaging system. While both inhibitory and stimulatory GPCR receptor components influence AC activity, desensitisation mechanisms may operate anomalously. The voltage gated calcium channels of L-, N- and P/Q-types appear affected (Hayashi et al. 2002). Hence this proposed mechanism may be crudely analogous to Lambert-Eaton syndrome (LES) and autoimmune impairment of calcium channels (Waterman 2001).

### Dysregulation of multiple complex biochemical pathways

Multiple complex biochemical pathways are mediated by AC-activating VNs. Some key pathways are considered below. Effector pathways for apoptosis are considered along with glutamate metabolism as a model for dysregulation of vital neurotransmitter and biochemical mechanisms postulated in VN auto-immune disorders. These pathways exemplify mechanisms operating in conditions of cellular and somatic stress within and beyond the central nervous system (Staines 2005a).

PACAP may be a potent mediator of the stress response to certain stimuli (Norrholm et al. 2005). Delgado (2002) reports inhibition of the MEKK1/MEK4/JNK pathway, leading to a reduction in phosphorylated c-Jun and stimulation of JunB, mediated through the VPAC1 receptor via the cAMP/PKA pathway. VIP/PACAP interference with the stress-induced SAPK/JNK pathway in activated microglia may thus represent a significant element in the regulation of inflammatory responses in the CNS by endogenous VNs.

Dysregulation of dehydroepiandrosterone (DHEA) metabolism has been noted in CFS (Maes et al. 2005, Cleare et al. 2004) and abnormal responses to ACTH occurs indicating inappropriate responses to stress (Scott et al. 2000). PACAP has a stimulatory effect on steroid secretion including cortisol and DHEAS in the adrenal gland and is mediated via catecholamines (Breault et al. 2000). VIP also is involved in ACTH-independent regulation of steroidogenesis in adrenal (tumour) cells (Haidan et al. 1998).

PACAP and VIP are potent neurotrophic substances and play a vital role in neuronal survival (Shioda et al. 1998). PACAP inhibits apoptosis in many tissues including cerebellar granule cells by inhibition of caspase-3, and mitochondrial pathways play a pivotal role in these anti-apoptotic effects. Ceramide (C2) mitochondrial potential inhibitory effects mediated via caspase systems together with cytochrome c release from mitochondria are countered via PACAP in apoptosis. PACAP acts by strongly inhibiting C2-ceramide-induced activation of caspase-3 (Vaudry et al. 2003, Falluel-Morel et al. 2004). Ceramide-induced apoptosis is also inhibited in PC12 cells by PACAP by affecting signaling downstream of JNK activation (Hartfield et al. 1998). Moreover, PACAP has been shown to stimulate MAPK in both PKA- and PKC-independent manner in astrocytes (Moroo et al. 1998). VIP also inhibits translocation of cytochrome c from mitochondria in hippocampal cells in protecting against apoptotic cell death (Antonawich and Said 2002). Hence, the implications for VN failure are serious as PACAP/VIP play a critical role in mitochondrial and other pathways in protecting against apoptosis.

Complex biochemical pathways intersect immune and neurotransmitter functions and are modulated by VNs and glutamate serves as a useful model. For example, exposure to ammonia during prenatal and lactation periods results in long-lasting impairment of *N*-methyl-D-aspartate (NMDA) receptor function which may be associated with altered aspartate amintotransferase activity (Minana et al. 1995) and hence altered glutamate function which may be relevant in SIDS.

Lee et al. (2005) note significant differences for alanine/aspartate transaminase and gamma glutamyl transaminase in blood tests in Gulf War veterans. NMDA is known to have a trophic effect on cerebellar granule cells (Caballero-Benitez et al. 2004) and is known to enhance activity of AAT considerably (Moran and Rivera-Gaxiola 1992). NMDA may in turn be modulated by cAMP which is induced by PACAP (Llansola et al. 2004). PACAP is able to enhance NMDA receptor function and also enable RACK1 expression of brain-derived neurotrophic factor (BDNF) (Yaka et al. 2003). Also, PACAP helps regulate glial glutamate transport and metabolism (Figiel and Engele 2000). Hence, loss or compromise of function of PACAP would be expected to have significant effects on NMDA and neuronal function.

Intra- and extra-cellular calcium regulation appears to be vital in VN function. Dziema and Obrietan (2002) note that PACAP potentiates L type Ca(2+) activity. Suprachiasmatic nucleus neurons become sensitive to glutamate only after PACAP administration, suggesting that PACAP sets the lower concentration threshold required for glutamate to initiate a robust rise in postsynaptic cytosolic Ca(2+). Modulatory actions of PACAP are related to the p42/44 mitogen activated protein kinase (MAPK) signal transduction cascade. Aoyagi and Takahashi (2001) note that PACAP enhances Ca(2+) dependent glutamate neurotransmitter release in PC12 cells by modulating steps subsequent to Ca(2+) entry and Chen et al. (2000) note that ATP increases Ca(2+) by mobilising internally stored Ca(2+) followed by an influx of Ca(2+). Defer et al. (2000) note that AC is tissue specific particularly in relation to Ca(2+)/-calmodulin functions and that signals received by GPCRs can be differentially integrated. Calcium regulated by VNs thus plays a key role in coordinating cellular and neurotransmission functions (Endoh 2004).

Receptor function including AC is vital in coordinated and integrated VN activity. Nowak and Zawiska (1999) note that the plethora of GPCRs and the functional differentiation of G-protein subunits and many AC isoforms permit a very complex signaling system with a wide variety of integrative characteristics. Chabardes et al. (1999) note AC types 5 and 6 constitute a sub-family having the property of being inhibited by submicromolar Ca2+ concentrations in addition to Galpha(i)-mediated processes. This ensures wide responses in cAMP synthesis. Mons et al. (1999) note AC types 1 and 8 stimulate Ca2+/calmodulin in the hippocampus and this suggests a role for hippocampus related memory function.

Chern (2000) notes that AC isoenzymes are tightly controlled by various signals and one of their most important impacts is on the complexity and fine-tuning of cellular signaling especially in the CNS where multiple signals constantly occur. MAP kinase and CREB mechanisms may also become disrupted resulting in significant neuro-physiological impairment. Hippocampal functions such as long-term potentiation by the mossy fibre pathway are likely to be associated with PACI-R in presynaptic cells (Otto et al. 1999) suggesting their vulnerability to VN dysfunction.

Shaked et al. (2005) note that T cells reactive to CNS-specific self-antigens protect neurons against glutamate toxicity. Antigen-specific autoimmune T cells increase the ability of microglia-enriched cultures to remove glutamate. This up-regulation of glutamate uptake induced by IFN-gamma activation is not accompanied by the acute inflammatory response seen in LPS-activated cultures. Hence, T cells or their cytokines can cause microglia to adopt a phenotype that facilitates rather than impairs glutamate clearance to contribute to restoration of homeostasis.

Rangon et al. (2005) note that VIP has a protective effect for glutamate acting via the VPAC2 receptor. Similarly, Shintani et al. (2005) note PACAP mRNA levels were increased up to $3.5 \times 8$ h after glutamate exposure in rat neuronal cultures indicating a neuroprotective role of PACAP. Moreover, others (Dong et al. 2000, Kopp et al. 2001) note the role of intracellular calcium regulation by PACAP as a mechanism to control glutamate toxicity in hippocampal and suprachiasmatic neurons. Glutamate transporters have a vital role in clearing glutamate from the extracellular environment and absorbing it via astrocytes to protect neurons from toxicity (Onoue et al. 2002) and these transporters are potently activated by PACAP (Schluter et al. 2002, Figiel et al. 2003).

As noted above, PACAP plays a critical role in protecting tissues from hypoxia. Rabl et al. (2002) note that turtles have much greater levels than mammals to protect them from diving induced hypoxia. The gaseous neurotransmitters NO and CO play vital roles in cellular metabolism and are tightly regulated by VNs to preserve homeostasis. These gaseous transmitters are postulated to be associated with SIDS because of their known association with cigarette smoking (Staines 2004b). Martinez et al. (2005) also note the role of the PAC1 receptor in NO signaling and septic shock. Hence, VNs have complex and crucial regulatory functions of gaseous neurotransmitters and may include NO, CO and ammonia.

Insulin activity is significantly modulated by VNs through AC and cyclic AMP/PKA pathways (Radosavljevic et al. 2004). PACAP potently enhances glucose-stimulated insulin secretion in pancreatic islets and enhances insulin action in adipocytes (Nakata et al. 1999). Hence, PACAP and VIP play a significant role in neuroendocrine regulation of insulin-glucose homeostasis (Wei and Mojsov 1996) and the PAC1 receptor is required to maintain normal insulin secretory responsiveness to glucose (Jamen et al. 2000). CFS patients display significantly lower ACTH response levels in stress testing and insulin tolerance tests (ITT) (Gaab et al. 2002) as well as reporting subjective hypoglycaemia. VN dysregulation may account for these problems (Shintani et al. 2003).

### CPG and heat shock proteins may have important roles in vasoactive neuropeptide immune dysregulation

Cytosine-guanine dinucleotide fragments (CpG) of DNA are postulated to be the active ingredients in bacterial extracts able to induce immune responses including adjuvant effects and may have applications in human vaccines (Krieg et al. 1995, Ada and Ramshaw 2003, Tsuchiya et al. 2005). The immune activating effects of CpG may occur through acquired bacterial or viral DNA, oligodeoxynucleotide (ODN) or through self-derived DNA fragments and may have a role in other autoimmune conditions such as systemic lupus erythematosus (SLE) (Krieg 1995, Jones et al. 2002, Januchowski et al. 2004). They rescue B cells from apoptosis, suggesting an autoimmune role (Yi et al. 1999).

Microbial pathogens containing CpG fragments are known to bind Toll-like receptors and/or stimulate microbe-specific T cells to express CD40 ligand, thereby licensing antigen presenting cells that bear both microbial and auto-antigens to break tolerance and precipitate autoimmune disease (Ichikawa et al. 2002, Ebert et al. 2005). In lupus-prone mice, abnormal innate responses through their pattern-recognition TLR9 receptors implies that response to infectious danger in these mice is inappropriate and may be linked to lupus pathogenesis (Krieg 1995, Lenert et al. 2003). Hence, autoimmune and inflammatory processes are known to be induced through these mechanisms.

HSPs may also be implicated in the recognition of bacterial or mammalian CpG DNA by acting as a ligand transfer molecule and/or play a central role in the signalling cascade induced by CpG DNA (Bandholtz et al. 2003). Moreover, innate and adaptive immune mechanisms may act through a cross priming adjuvant mechanism to engage heat shock protein in autoreactive responses (Kumaraguru et al. 2003). HSPs also activate Toll-like receptors in triggering innate immunity, perhaps through adjuvant-like signals (van Eden et al. 2003, Millar et al. 2003).

HSPs thus have an established place in regulation of the immune response (Pockley 2003). HSPs also bind with other antigenic peptides to form immunostimulatory complexes (Srivistava 2002) and interestingly may take the role of antigenic presentation and processing in immunoprotected regions such as the central nervous system (Oglesbee et al. 2002). Indeed aberrant self HSP expression may lead to enhancement/modulation of autoimmune responses in the context of myelin basic protein and MHC class II type interactions (Mycko et al. 2004).

As noted above mammalian DNA normally has lower than predicted CpG dinucleotide fragments and these are also usually methylated (Shiota 2004). These characteristics differ from bacterial and viral DNA

which contains higher percentages of CpG fragments and these are more likely to be hypomethylated, providing a biological "friend or foe" identification system.

Ancient DNA sequences mimicking bacterial and viral genomes containing higher proportions of CpG elements have become incorporated into mammalian DNA as human endogenous retrovirus (HERV). These genetic components have become methylated over time making them mostly benign components of mammalian DNA. However, these DNA components may undergo hypomethylation through a range of stimulating factors, making them able to regulate transcriptional activity and expression of the HERV family (Lavie et al. 2005) with implications for a range of pathologies. Interestingly promoter regions of the secretin receptor gene have high CpG representation (Lee et al. 2004) which may also be a feature of PACAP and VIP receptor genes.

Spontaneous hypomethylation of susceptible endogenous CpG sequences, or exposure to exogenous bacterial CpG DNA and subsequent stimulation of cellular processes may mediate innate and acquired immune pathways including class switching from IgM to more pathogenic IgG immunoglobulin types (He et al. 2004). IgM and IgG reactivity to key fragments of certain VNs or related HSPs thus might theoretically occur. Such postulated mechanisms could establish perverse autoreactive loss of immunological tolerance and effectively create immunisation against these VNs. The known susceptibility of CpG fragments to hypomethylation from toxic causes such as biological poisons and radiation might predispose to the development of these and other pathologies (Chen et al. 2004, Pogribny et al. 2004). These mechanisms might also link fatigue-related VN autoimmune disorders to exposure to radiological, biological and chemical warfare agents, extending the context of public health and military medicine importance of these postulated disorders.

### Therapeutic and preventive interventions

The development of therapeutic and preventive strategies for postulated VN autoimmune conditions will be determined by the complex pathophysiology underpinning them. Mostly these strategies relate to restoring the functional characteristics of VNs possibly compromised (Staines 2005c). Analogies with other illnesses may provide therapeutic parallels. Some speculative possibilities are listed below:

### *Substitution/replacement*

Simple substitution/replacement interventions are the most obvious, however, a significant theoretical impediment is the propensity of these substances to induce tachyphylaxis (Whalen et al. 1999). Feedback signaling of VNs is thought to be quite complex and cascade effects are still largely unpredictable. Catalytic antibody self-regulatory activity occurs for VNs, although little is known about how extensively self-regulation occurs. Catalytic activity of these antibodies has been described as an innate function originating over the course of phylogenetic evolution as opposed to somatic processes (Gololobov et al. 1999).

Because of rapid natural degradation of VNs, e.g. by naturally occurring antibodies and hydrolysis, liposomal therapeutic vehicles are being explored to prolong their biological effects. Sterically stabilised liposomes (SSL) are being developed to provide long acting formulations of VIP resistant to the rapid degradation usually observed (Sethi et al. 2005).

### *Plasma exchange*

Perverse immunological memory in early B cell clones may also be an important factor in establishing basic autoimmune pathology. While traditionally viewed as the line of humoral defence, B cell activation is increasingly being associated with T cell lineage development and cellular immune responses. Hence, B cells as therapeutic targets have increasing potential in autoimmune disorders (Looney et al. 2004). Specific mono- and polyclonal catalytic antibody generation may provide opportunities for targeting abnormal autoimmune epitopes in treatment. Circulating T and B cells and immunoglobulins could be filtered and pathogenic cells and antibodies removed. This may provide a screening and potentially therapeutic approach to prevent SIDS and other fatigue-related disorders, but is not established.

### *Analogues with MG treatment*

The known association of VPAC2 receptors with acetylcholine and muscle function (Hinkle et al. 2005) suggests a patho-mechanism crudely analogous with autoimmune dysfunction in MG and may provide a useful model to explore. Hence treatment options such as pyridostigmine and thymectomy may be considered. In a series of three case reports, Kawamura et al. (2003) describe successful use of oral pyridostigmine in the treatment of CFS. This is an interesting finding given the possible association of pyridostigmine with the aetiology of GWS (Abou-Donia et al. 2004, Staines 2005b). Should specific anti-VN T cells from thymus prove to be associated with GWS and CFS, thymectomy could be considered. However, given that antigen in GWS and CFS may indeed be VNs or their receptors and related HSPs widely distributed throughout the body such a proposed solution does not immediately appear rational. Corticosteroids are used judiciously with MG but may not yet be justified in postulated VN autoimmune fatigue-related conditions.

*Analogues with LES treatment*

Should calcium channelopathy prove to be a significant element in VN autoimmune disorders, parallels with LES may prove useful. Hence calcium channel promoters, acetylcholinesterases, immune suppressants, plasma exchange and intravenous immunoglobulins may be considered in this context.

*Anti-idiotype antibodies*

Should fatigue-related disorders be found to result from immune responses to VNs or their receptors there may be scope to develop anti-idiotype antibodies. While theoretically a possibility, there are no close analogues of these disorders in which this treatment is effective. Because of the known association of the VPAC2 receptor with acetylcholine activity as noted above, MG may be a crude analogue inasmuch as acetylcholine receptors are compromised albeit by a different mechanism. However, anti-idiotype antibody treatment in MG is of equivocal effectiveness. Alternatively should self antibodies to VN regulatory mechanisms be the causative mechanism these could be extracted by plasma exchange or specific anti-idiotype antibodies.

*Epigenetic DNA modifications*

T- and B-cell functioning may be influenced by VNs through selective screening of specific epitope carriers. While this research is still in its infancy, potentially VN analogous autoimmune conditions such as SLE may shed light on new CpG therapeutic technologies (Januchowski et al. 2004). Targeting genomic expression abnormalities may also be possible through therapeutic CpG immunostimulating and immunosuppressive technologies. Genomic modification of VN expression may be relevant in SIDS should respiratory control deficits be proven to play a causal role. Moreover, CpG binding proteins in microglia are mostly RNA processing enzymes suggesting a profound array of opportunities by which CpG may influence cellular processes in the CNS (Zhang et al. 2005).

*CpG and DNA vaccines*

Prevention of possible VN autoimmune fatigue related disorders may be important areas for future development. Immunoprotective CpG DNA fragments may be applied as vaccines to protect VNs or their receptors from degradation or dysfunction. Beneficial natural immunity against pro-inflammatory cytokines may also be amplified by DNA vaccines (Karin 2004) although these will need to be developed with care (Klinman et al. 2003). Strategies to prevent or treat VN autoimmune disorders may include active and passive vaccination to protect VN receptors from direct immunogenic or indirect molecular mimicry effects. The underlying principle for vaccination would be to protect those at risk from neurological autoimmune dysfunction. However, this approach would be complex and criteria for being at risk would need to be identified. To speculate, a vaccine for SIDS may be theoretically possible based on protecting vital VN function from disruption at critical stages in infant development.

*Novel neuroprotective agents*

Novel small peptides with stress-protein-like sequences have been identified which exhibit strong levels of neuroprotection. These proteins protect neurons from cell death associated with electrical activity and heat shock protein antibodies and, therefore, may play a role in the treatment of neurodegenerative diseases. Therapeutic strategies for a range of neuroprotective substances including members of the VIP/PACAP family are also suggested (Brenneman and Gozes 1996, Gozes and Divinski 2004).

Preservation of function by other substances regulated by VNs may also be considered. For example, CSF endogenous opioid substances are regulated by PACAP and these have a role in cerebral arterial responses to hypoxia (Wilderman and Armstead 1997), which in turn may be relevant to protection from SIDS. Endogenous pain mediation may play a significant role in these conditions and restoration of this function may be appropriate if shown to be attributed to VN dysfunction (Julien et al. 2005). Finally, mutually enhancing "cross-over" effects of VIP/PACAP functioning may suggest that therapeutic interventions may have synergistic benefits with these substances (Samborski et al. 2004).

*Drug treatments*

Pharmaceuticals such as anti-depressants have been shown empirically to provide symptomatic relief to some fatigue-related disorders. These conditions have been shown not to be primary organic depression but may be explained as collateral symptomatology to possible VN autoimmune dysfunction.

Blockade of 5-HT could elicit symptomatology consistent with a VN autoimmune disorders. This effect may be explained by the role of serotonin in controlling VIP release mechanisms. Altered VIP expression may occur prenatally through serotonin imprinting in ontogenesis (Mirochnik et al. 2003) suggesting implications for monitoring the use of selective serotonin uptake inhibitor (SSRI) and tricyclic anti-depressants in pregnancy. However, decreased 5-HT1A receptor numbers and affinity are noted in CFS particularly in the hippocampus (Cleare et al. 2005) possibly indicating heterogenous modulating relationships between VIP and 5-HT receptors in CFS. Chloroquine may also have a role in treatment or prevention (Hong et al. 2004).

*Phosphodiesterase (PDE) inhibitors*

Because of the likely cAMP disruption in these conditions, cAMP promoting agents such as phosphodiesterase inhibitors may have a role (Staines 2006). Drugs such as rolipram, a phosphodiesterase type 4 inhibitor, activate cAMP-response element binding proteins (CREB) signalling as well as enhancing cAMP levels by impeding cAMP catabolism (Conti and Blendy 2004). Imipramine also appears to have a key role in cAMP metabolism and, therefore, may be useful in combination drug therapy (Itoh et al. 2004, Knuuttila et al. 2004). Rolipram was developed as an anti-depression drug but has been found also to have anti-inflammatory and immunoregulatory activities (Sommer et al. 1995, Abbas et al. 2000). Unfortunately, side-effects such as nausea, vomiting and headache are reported suggesting the need for less side effect-inducing analogues in therapy (Xu et al. 1999) and continuous administration may be necessary to sustain its therapeutic effect (Martinez et al. 1999). Later-generation drugs of this family may prove to have better tolerance.

### Conclusion

The autoimmune hypothesis of VNs suggests that relatively minor infection or inflammation results in predictable pro-inflammatory cytokine and other responses which may have subsequent serious effects involving VN dysfunction. Other pro-inflammatory effects such as NO release and possible chemical sensitivities may also result. Modulation and termination of these inflammatory responses is required by VNs. Autoimmune effects, e.g. on PACAP/VIP or the PAC1/VPAC1/VPAC2 receptors will have a negating effect on VN function and also subsequent effects on intracellular mechanisms.

While some inflammatory or infectious events may be trivial, compromise of the functions of VNs such as PACAP/VIP/CGRP is not. Brain, cardiac and other organs known to exhibit similar PACAP/VIP receptor function would also be expected to demonstrate dysfunction somewhat simultaneously. Prevention of SIDS and other disorders if shown to be VN autoimmune conditions may evolve from these concepts.

Public health implications may exist if “epidemics” or simply seasonal circulating organisms have particular molecular mimicry with VNs or their receptors. Short term relatively benign IgM may shift to a more pathogenic IgG phenotype as autoimmune responses to VNs/receptors and result in longer-term profound impairment and disability. These VN autoimmune processes may also have implications for military medicine where radiological, chemical and biological agents may play an important role in pathogenesis.

Postulated autoimmune VN conditions may share a common pathophysiology in contributing to apoptosis of neuronal and other vital neurological cells and that this underpins failure or compromise of important neuroregulatory mechanisms. Perhaps paradoxically, necessary apoptosis of autoreactive immunological cells, e.g. B and plasma cells may not occur and this predisposes to autoimmune dysfunction of VNs or their receptors. Dysregulation of innate immune systems through CpG and TLR9 interactions may also prove to have important roles in establishing autoreactivity.

Further understanding of possible autoimmune dysfunction of these VNs and their receptors may elucidate the mechanisms of disabling fatigue-related syndromes such as CFS and GWS, and possibly SIDS, and open the way for routine laboratory investigations and prevention options. VN and receptor reactivation may prove to become successful interventions. A spectrum of interventions including genomic, immunological and biochemical/drug therapies may prove to be possible in VN autoimmune fatigue-related disorders. Interventions such as phosphodiesterase inhibitors, immunotherapy, VN replacement or VN receptor reactivation may prove to be useful in these conditions but are not yet tested.

### References

Abbas N, Zou LP, Pelidou SH, Winblad B, Zhu J. 2000. Protective effect of Rolipram in experimental autoimmune neuritis: Protection is associated with down-regulation of IFN-gamma and inflammatory chemokines as well as up-regulation of IL-4 in peripheral nervous system. Autoimmunity 32(2):93–99.

Abou-Donia MB, Dechkovskaia AM, Goldstein LB, Abdel-Rahman A, Bullman SL, Khan WA. 2004. Co-exposure to pyridostigmine bromide, DEET, and/or permethrin causes sensorimotor deficit and alterations in brain acetylcholinesterase activity. Pharmacol Biochem Behav 77(2):253–262.

Ada G, Ramshaw I. 2003. DNA vaccination. Expert Opin Emerg Drugs 8(1):27–35.

Aino H, Hashimoto H, Ogawa N, Nishino A, Yamamoto K, Nogi H, Nagata S, Baba A. 1995. Structure of the gene encoding the mouse pituitary adenylate cyclase-activating polypeptide receptor. Gene 164(2):301–304.

Antonawich FJ, Said SI. 2002. Vasoactive intestinal peptide attenuates cyctochrome c translocation, and apoptosis, in rat hippocampal stem cells. Neurosci Lett 325(3):151–154.

Aoyagi K, Takahashi M. 2001. Pituitary adenylate cyclase-activating polypeptide enhances Ca(2+)-dependent neurotransmitter release from PC12 cells and cultured cerebellar granule cells without affecting intracellular Ca(2+) mobilisation. Biochem Biophys Res Commun 286(3):646–651.

Arimura A. 1998. Perspectives on pituitary adenylate cyclase-activating polypeptide (PACAP) in the neuroendocrine, endocrine and nervous systems. Jpn J Physiol 48(5):301–331.

Arnsten AF, Li BM. 2005. Neurobiology of executive functions: Catecholamine influences on prefrontal cortical functions. Biol Psychiatry 57(11):1377–1384.

Bandholtz L, Guo Y, Palmberg C, Mattson K, Ohlsson B, High A, Shabanowitz J, Hunt DF, Jomvall H, Wigzell H, Agerberth B, Gudmundsson GH. 2003. Heat shock protein binds CpG oligonucleotides directly: Implications for hsp90 as a missing link in CpG signalling and recognition. Cell Mol Life Sci 60(2):422–429.

Bangale Y, Cavill D, Gordon T, Planque S, Taguchi H, Bhatia G, Nishiyama Y, Arnett F, Paul S. 2002. Vasoactive intestinal peptide binding autoantibodies in autoimmune humans and mice. Peptides 23(12):2251–2257.

Bedoui S, Miyake S, Lin Y, Miyamoto K, Oki S, Kawamura N, Beck-Sickinger A, von Horsten S, Yamamura T. 2003. Neuropeptide Y (NPY) suppresses experimental autoimmune encephalomyelitis: NPY1 receptor-specific inhibition of autoreactive Th1 responses *in vivo*. J Immunol 171(7):3451–3458.

Bhave SV, Hoffman PL. 2004. Phosphatidylinositol 3′-OH kinase and protein kinase A pathways mediate the anti-apoptotic effect of pituitary adenylyl cyclase-activating polypeptide in cultured cerebellar granule neurons: Modulation by ethanol. J Neurochem 88(2):359–369.

Breault L, Yon L, Montero M, Chouinard L, Contesse V, Delarue C, Fournier A, Lehoux JG, Vaudry H, Gallo-Payet N. 2000. Occurrence and effect of PACAP in the human fetal adrenal gland. Ann N Y Acad Sci 921:429–433.

Brenneman DE, Gozes I. 1996. A femtomolar-acting neuroprotective peptide. J Clin Invest 97(10):2299–2307.

Broad PM, Symes AJ, Thakker RV, Craig RK. 1989. Structure and methylation of the human calcitonin/alpha-CGRP gene. Nucleic Acids Res 17(17):6999–7011.

Buffelli M, Pasino E, Cangiano A. 2001. *In vivo* acetylcholine receptor expression induced by calcitonin gene-related peptide in rat soleus muscle. Neuroscience 104(2):561–567.

Caballero-Benitez A, Alavez S, Uribe RM, Moran J. 2004. Regulation of glutamate synthesising enzymes by NMDA and potassium in cerebellar cells. Eur J Neurosci 19(8):2030–2038.

Cao YJ, Gimpl G, Fahrenholz F. 2000. A mutation in the second intracellular loop of the pituitary adenylate cyclase activating polypeptide type I receptor confers constitutive receptor activation. FEBS Lett 469(2/3):142–146.

Cauli B, Tong XK, Rancillac A, Serluca N, Lambolez B, Rossier J, Hamel E. 2004. Cortical GABA interneurons in neurovascular coupling: Relays for subcortical vasoactive pathways. J Neurosci 24(41):8940–8949.

Chabardes D, Imbert-Teboul M, Elalouf JM. 1999. Functional properties of Ca2 + -inhibitable type 5 and 6 adenylyl cyclases and role of Ca2 + increase in the inhibition of intracellular cAMP content. Cell Signal 11(9):651–663.

Chang Y, Lawson LJ, Hancock JC, Hoover DB. 2005. Pituitary adenylate cyclase-activating polypeptide: Localisation and differential influence on isolated hearts from rats and guinea pigs. Regul Pept 129(1/3):139–146.

Chelbi-Alix MK, Boissard C, Sripati CE, Rosselin G, Thang MN. 1991. Vip induces in HT-29 cells 2′5′oligoadenylate synthetase and antiviral state via interferon beta/alpha synthesis. Peptides 12(5):1085–1093.

Chen H, Li S, Liu J, Diwan BA, Barrett JC, Waalkes MP. 2004. Chronic inorganic arsenic exposure induces hepatic global and individual gene hypomethylation: Implications for arsenic hepatocarcinogenesis. Carcinogenesis 25(9):1779–1786.

Chen L, Maruyamam D, Sugiyama M, Sakai T, Mogi C, Kato M, Kurotani R, Shirasawa N, Takaki A, Renner U, Kato Y, Inoue K. 2000. Cytological characterisation of a pituitary folliculo-stellate-like cell line, Tpit/F1, with special reference to adenosine triphosphate-mediated neuronal nitric oxide synthase expression and nitric oxide secretion. Endocrinology 141(10):3603–3610.

Chern Y. 2000. Regulation of adenylyl cyclase in the central nervous system. Cell Signal 12(4):195–204.

Cleare AJ, Messa C, Rabiner EA, Grasby PM. 2005. Brain 5-HT1A receptor binding in chronic fatigue syndrome measured using positron emission tomography and [11C]WAY-100635. Biol Psychiatry 57(3):239–246.

Cleare AJ, O'Keane V, Miell JP. 2004. Levels of DHEA and DHEAS and responses to CRH stimulation and hydrocortisone treatment in chronic fatigue syndrome. Psychoneuroendocrinology 29(6):724–732.

Cohen G, Gressens P, Gallego J, Gaulter C. 2002. Depression of hypoxic arousal response in adolescent mice following antenatal vasoactive intestinal polypeptide blockade. J Physiol 540(Pt2): 691–699.

Conti AC, Blendy JA. 2004. Regulation of antidepressant activity by cAMP response element binding proteins. Mol Neurobiol 30(2):143–155.

Cummings KJ, Pendlebury JD, Sherwood NM, Wilson RJ. 2004. Sudden neonatal death in PACAP-deficient mice is associated with reduced respiratory chemoresponse and susceptibility to apnoea. J Physiol 555(Pt1):15–26.

Dautzenberg FM, Hauger RL. 2001. G-protein-coupled receptor kinase 3- and protein kinase C-mediated desensitisation of the PACAP receptor type 1 in human Y-79 retinoblastoma cells. Neuropharmacology 40(3):394–407.

Defer N, Best-Belpomme M, Hanoune J. 2000. Tissue specificity and physiological relevance of various isoforms of adenylyl cyclase. Am J Physiol Renal Physiol 279(3):F400–F416.

DeHaven WI, Cuevas J. 2004. VPAC receptor modulation of neuroexcitability in intracardiac neurons: Dependence on intracellular mobilization and synergistic enhancement by PAC1 receptor activation. J Biol Chem 279(39):40609–40621.

Delgado M, Abad C, Martinez C, Juarranz MG, Arranz A, Gomariz RP, Leceta J. 2002. Vasoactive intestinal peptide in the immune system: Potential therapeutic role in inflammatory and autoimmune diseases. J Mol Med 80(1):16–24.

Delgado M. 2002. Vasoactive intestinal peptide and pituitary adenylate cyclase activating polypeptide inhibit the MEKK1/MEK4/JNK signalling pathway in endotoxin-activated microglia. Biochem Biopys Res Commun 293(2):771–776.

Delgado M, Gonzalez-Rey E, Ganea D. 2005. The neuropeptide vasoactive intestinal peptide generates tolerogenic dendritic cells. J Immunol 175(11):7311–7324.

Dohi K, Mizushima H, Nakajo S, Ohtaki H, Matsunaga S, Aruga T, Shioda S. 2002. Pituitary adenylate cyclase-activating polypeptide (PACAP) prevents hippocampal neurons from apoptosis by inhibiting JNK/SAPK and p38 signal transduction pathways. Regul Pep 109(1/3):83–88.

Dong Y, Tang TS, Lu CL, He C, Dong JB, Huang XY, Sun FZ, Bao X. 2000. Pituitary adenylate cyclase activating polypeptide ameliorates the damage and inhibits the increase of intracellular calcium concentration in cultured hippocampal neurons induced by glutamate. Sheng Li Xue Bao 52(5):402–406.

Dvorakova MC, Pfeil U, Kuncova J, Sviglerova J, Galvis G, Krasteva G, Konig P, Grau V, Slavikova J, Kummer W. 2005. Down-regulation of vasoactive intestinal peptide and altered expression of its receptors in rat diabetic cardiomyopathy. Cell Tissue Res 13:1–11, [Epub ahead of print].

Dziema H, Obrietan K. 2002. PACAP potentiates L-type calcium channel conductance in suprachiasmatic nucleus neurons by activating the MAPK pathway. J Neurophysiol 88(3): 1374–1386.

Ebert S, Gerber J, Bader S, Muhlhauser F, Brechtel K, Mitchell TJ, Nau R. 2005. Dose-dependent activation of microglial cells by Toll-like receptor agonists alone and in combination. J Neuroimmunol 159(1–2):87–96.

Endoh T. 2004. Modulation of voltage-dependent calcium channels by neurotransmitters and neuropeptides in parasympathetic submandibular ganglion neurons. Arch Oral Biol 49(7): 539–557.

Fahrenkrug J, Hannibal J, Tams J, Georg B. 2000. Immunohistochemical localisation of the VIP1 receptor (VPAC1R) in rat cerebral blood vessels: Relation to PACAP and VIP containing fibres. J Cereb Blood Flow Metab 20(8):1205–1214.

Fahrenkrug J, Hannibal J. 2004. Neurotransmitters co-existing with VIP or PACAP. Peptides 25(3):393–401.

Falluel-Morel A, Aubert N, Vaudry D, Basille M, Fontaine M, Fournier A, Vaudry H. 2004. Opposite regulation of the mitochondrial apoptotic pathway by C2-ceramide and PACAP through a MAP-kinase-dependent mechanism in cerebellar granule cells. J Neurochem 91(5):1231–1243.

Figiel M, Engele J. 2000. Pituitary adenylate cyclase-activating polypeptide (PACAP), a neuron-derived peptide regulating glial glutamate transport and metabolism. J. Neurosci 20(10): 3596–3605.

Figiel M, Maucher T, Rozyczkz J, Bayatti N, Engele J. 2003. Regulation of glial glutamate transporter expression by growth factors. Exp Neurol 183(1):124–135.

Gaab J, Huster D, Peisen R, Engert V, Heitz V, Schad T, Schurmeyer TH, Ehlert U. 2002. Hypothalamic-pituitary-adrenal axis reactivity in chronic fatigue syndromes and health under psychological, physiological, and pharmacological stimulation. Psychosom Med 64(6):951–962.

Ganea D, Rodriguez R, Delgado M. 2003. Vasoactive intestinal peptide and pituitary adenylate cyclase-activating polypeptide: Players in innate and adaptive immunity. Cell Mol Biol (Noisy-le-grand) 49(2):127–142.

Goetzl EJ, Wang W, McGiffert C, Huang MC, Graler MH. 2004. Sphingosine 1-phosphate and its G-protein-coupled receptors constitute a multifunctional immunoregulatory system. J Cell Biochem 92(6):1104–1114.

Goldberg B, Urnovitz HB, Stricker RB. 2000. Beyond danger: Unmethylated CpG dinucleotides and the immunopathogenesis of disease. Immunol Lett 73(1):13–18.

Gololobov G, Sun M, Paul S. 1999. Innate antibody catalysis. Mol Immunol 36(18):1215–1222.

Gordon TP, Bolstad AI, Rischmueller M, Jonsson R, Waterman SA. 2001. Autoantibodies in primary Sjogren's syndrome: New insights into mechanisms of autoantibody diversification and disease pathogenesis. Autoimmunity 34(2):123–132.

Gozes I, Divinski I. 2004. The femtomolar-acting NAP interacts with microtubules: Novel aspects of astrocyte protection. J Alzheimers Dis (6 Suppl):S37–S41.

Gray SL, Yamaguchi N, Vencova P, Sherwood NM. 2002. Temperature-sensitive phenotype in mice lacking pituitary adenylate cyclase-activating polypeptide. Endocrinology 143(10):3946–3954.

Haidan A, Hilbers U, Bornstein SR, Ehrhart-Bornstein M. 1998. Human adrenocortical NCI-H295 cells express VIP receptors. Steroidogenic effects of vasoactive intestinal peptide (VIP). Peptides 19(9):1511–1517.

Halls ML, Bathgate RA, Summers RJ. 2005. Signal switching after stimulation of LGR7 receptors by human relaxin 2. Ann N Y Acad Sci 1041:288–291.

Hamelink C, Tjurmina O, Damadzic R, Young WS, Weihe E, Lee HW, Eiden LE. 2002. Pituitary adenylate cyclase-activating polypeptide is a sympathoadrenal neurotransmitter involved in catecholamine regulation and glucohomeostasis. Proc Natl Acad Sci USA 99(1):461–466.

Han JS, Li W, Neugebauer V. 2005. Critical role of calcitonin gene-related peptide 1 receptors in the amygdala in synaptic plasticity and pain behavior. J Neurosci 25(46):10717–10728.

Hannibal J. 2002. Pituitary adenylate cyclase-activating peptide in the rat central nervous system: An immunohistochemical and in situ hybridisation study. J Comp Neurol 453(4):389–417.

Hartfield PJ, Bilney AJ, Murray AW. 1998. Neurotrophic factors prevent ceramide-induced apoptosis downstream of c-Jun *N*-terminal kinase activation in PC12 cells. J Neurochem 71(1):161–169.

Hashimoto H. 2002. Physiological significance of pituitary adenylate cyclase-activating polypeptide (PACAP) in the nervous system. Yakugaku Zasshi 122(12):1109–1121.

Hayashi K, Endoh T, Shibukawa Y, Yamamoto T, Suzuki T. 2002. VIP and PACAP inhibit L-, N-, and P/Q-type Ca2 + channels of parasympathetic neurons in a voltage independent manner. Bull Tokyo Dent Coll 43(1):31–39.

He B, Qiao X, Cerutti A. 2004. CpG DNA induces IgG class switch DNA recombination by activating human B cells through an innate pathway that requires TLR9 and cooperates with IL-10. J Immunol 173(7):4479–4491.

Hinkle RT, Donnelly E, Cody DB, Sheldon RJ, Isfort RJ. 2005. Activation of the vasoactive intestinal peptide 2 receptor modulates normal and atrophying skeletal muscle mass and force. J Appl Physiol 98(2):655–662.

Hirose M, Leatmanoratn Z, Laurita KR, Carlson MD. 2001. Mechanism for pituitary adenylate cyclase-activating polypeptide-induced atrial fibrillation. J Cardiovasc Electrophysiol 12(12):1381–1386.

Holz GG, Habener JF. 1998. Black widow spider alpha-latroxin: A presynaptic neurotoxin that shares structural homology with the glucagon-like peptide-1 family of insulin secretagogic hormones. Comp Biochem Physiol B Biochem Mol Biol 121(2):177–184.

Hong Z, Jiang Z, Liangxi W, Guofu D, Ping L, Yongling L, Wendong P, Minghai W. 2004. Chloroquine protects mice from challenge with CpG ODN and LPS by decreasing proinflammatory cytokine release. Int Immunopharmacol 492:223–234.

Ichikawa HT, Williams LP, Segal BM. 2002. Activation of APCs through CD40 or Toll-like receptor overcomes tolerance and precipitates autoimmune disease. J Immunol 169(5): 2781–2787.

Iliev AI, Stringaris AK, Nau R, Neumann H. 2004. Neuronal injury mediated via stimulation of microglial toll-like receptor-9 (TLR9). FASEB J 18(2):412–414.

Ishizuka Y, Kashimoto K, Mochizuki T, Sato K, Ohshima K, Yanaihara N. 1992. Cardiovascular and respiratory actions of adenylate cyclase-activating polypeptides. Regul Pept 40(1):29–39.

Itkes AV. 1994. Oligoadenylate and cyclic AMP: Interrelation and mutual recognition. Prog Mol Subcell Biol 14:209–221.

Itoh T, Tokukura M, Abe K. 2004. Effects of rolipram, a phosphodiesterase 4 inhibitor, in combination with imipramine on depressive behaviour, CRE-binding activity and BDNF level in learned helplessness rats. Eur J Pharmacol 498(1/3):135–142.

Jamen F, Persson K, Bertrand G, Rodriguez-Henche N, Puech R, Bockaert J, Ahren B, Brabet P. 2000. PAC1 receptor-deficient mice display impaired insulinotropic response to glucose and reduced glucose tolerance. J Clin Invest 105(9):1307–1315.

Januchowski R, Prokop J, Jagodzinski PP. 2004. Role of epigenetic DNA alterations in the pathogenesis of systemic lupus erythematosus. J Appl Genet 45(2):237–248.

Johnson MC, McCormack RJ, Delgado M, Martinez C, Ganea D. 1996. Murine T-lymphocytes express vasoactive intestinal peptide receptor 1 (VIP-R1) mRNA. J Neuroimmunol 68(1/2):109–119.

Jones DE, Palmer JM, Burt AD, Walker C, Robe AJ, Kirby JA. 2002. Bacterial motif DNA as an adjuvant for the breakdown of immune self-tolerance to pyruvate dehydrogenase complex. Hepatology 36(3):679–686.

Julien N, Goffaux P, Arsenault P, Marchand S. 2005. Widespread pain in fibromyalgia is related to a deficit of endogenous pain inhibition. Pain 114(1/2):295–302.

Karin N. 2004. Induction of protective therapy for autoimmune diseases by targeted DNA vaccines encoding pro-inflammatory cytokines and chemokines. Curr Opin Mol Ther 6(1):27–33.

Kato H, Ito A, Kawanokuchi J, Jin S, Mizuno T, Ojika K, Ueda R, Suzumura A. 2004. Pituitary adenylate cyclase-activating polypeptide (PACAP) ameliorates experimental autoimmune encephalomyelitis by suppressing the functions of antigen presenting cells. Mult Scler 10(6):651–659.

Kawamura Y, Kihara M, Nishimoto K, Taki M. 2003. Efficacy of a half dose of oral pyridostigmine in the treatment of chronic fatigue syndrome: Three case reports. Pathophysiology 9(3):189–194.

Keino H, Kezuka T, Takeuchi M, Yamakawa N, Hattori T, Usui M. 2004. Prevention of experimental autoimmune uveoretinitis by vasoactive intestinal peptide. Arch Ophthalmol 122(8): 1179–1184.

Klinman DM, Zeuner R, Yamada H, Gursel M, Currie D, Gursel I. 2003. Regulation of CpG-induced immune activation by suppressive oligodeoxynucleotides. Ann N Y Acad Sci 1002: 112–123.

Knuuttila JE, Toronen P, Castren E. 2004. Effects of antidepressant drug imipramine on gene expression in rat prefrontal cortex. Neurochem Res 29(6):1235–1244.

Kong WJ, Scholtz AW, Kammen-Jolly K, Gluckert R, Hussl B, von Cauvenberg PB, Schrott-Fischer A. 2002. Ultrastructural evaluation of calcitonin gene-related peptide immunoreactivity in the human cochlea and vestibular endorgans. Eur J Neurosci 15(3):487–497.

Kopp MD, Meissl H, Dehghani F, Korf HW. 2001. The pituitary adenylate-cyclase activating polypeptide modulates glutamatergic calcium signalling: Investigations on rat suprachiasmatic nucleus neurons. J Neurochem 79(1):161–171.

Krieg AM, Yi AK, Matson S, Waldschmidt TJ, Bishop GA, Teasdale R, Koretzky GA, Klinman DM. 1995. CpG motifs in bacterial DNA trigger direct B-cell activation. Nature 374(6522):546–549.

Krieg AM. 1995. CpG DNA: A pathogenic factor in systemic lupus erythematosus. J Clin Immunol 15(6):284–292.

Krieg AM. 2003. CpG DNA: Trigger of sepsis, mediator of protection, or both? Scand J Infect Dis 35(9):653–659.

Kumaraguru U, Pack CD, Rouse BT. 2003. Toll-like receptor ligand links innate and adaptive immune responses by the production of heat-shock proteins. J Leukoc Biol 73(5):574–583.

Lavie L, Kitova M, Maldener E, Meese E, Mayer L. 2005. CpG methylation directly regulates transcriptional activity of the human endogenous retrovirus family HERV-K (HML-2). J Virol 79(2):876–883.

Lee HA, Bale AJ, Gabriel R. 2005. Results of investigations of Gulf War veterans. Clin Med 5(2):166–172.

Lee LT, Tan-Un KC, Pang RT, Lam DT, Chow BK. 2004. Regulation of the human secretin gene is controlled by the combined effects of CpG methylation, Sp1/Sp3 ratio, and the E-box element. Mol Endocrinol 18(7):1740–1755.

Lenert P, Goeken A, Handweger BS, Asman RF. 2003. Innate immune responses in lupus-prone Palmerston North mice: Differential responses to LPS and bacterial DNA/CpG oligonucleotides. J Clin Immunol 23(3):202–213.

Llansola M, Sanchez-Perez AM, Montoliu C, Felipo V. 2004. Modulation of NMDA receptor function by cyclic AMP in cerebellar neurones in culture. J Neurochem 91(3):591–599.

Looney RJ, Anolik J, Sanz I. 2004. B cells as therapeutic targets for rheumatic diseases. Curr Opin Rheumatol 16(3):180–185.

Lutz EM, Shen S, Mackay M, West K, Harmar AJ. 1999. Structure of the human VIPR2 gene for vasoactive intestinal peptide receptor type 2. FEBS Lett 458(2):197–203.

Lyon MJ, Payman RN. 2000. Comparison of the vascular innervation of the rat cochlea and vestibular system. Hear Res 141(1/2):189–198.

Maes M, Mihaylova I, De Ruyter M. 2005. Decreased dehydroepiandrosterone sulfate but normal insulin-like growth factor in Chronic Fatigue Syndrome (CFS): Relevance for the inflammatory response in CFS. Neuro Endocrinol Lett 26(5): 487–492.

Martinez C, Juarranz Y, Abad C, Arranz A, Miguel BG, Rosignoli F, Receta J, Gomariz RP. 2005. Analysis of the role of the PAC1 receptor in neutrophil recruitment, acute-phase response, and nitric oxide production in septic shock. J Leukoc Biol 77(5): 729–738.

Martinez I, Puerta C, Redondo C, Garcia-Merino A. 1999. Type IV phosphodiesterase inhibition in experimental allergic encephalomyelitis of Lewis rats: Sequential gene expression analysis of cytokines, adhesion molecules and the inducible nitric oxide synthase. J Neurol Sci 164(1):13–23.

Millar DG, Garza KM, Odermatt B, Elford AR, Ono N, Li Z, Ohashi PS. 2003. Hsp70 promotes antigen-presenting cell function and converts T-cell tolerance to autoimmunity *in vivo*. Nat Med 9(12):1465–1466.

Milligan G. 2003. Constitutive activity and inverse agonists of G-protein coupled receptors: A current perspective. Mol Pharm 64(6):1271–1276.

Minana MD, Marcaida G, Grisolia S, Felipo V. 1995. Prenatal exposure of rats to ammonia impairs NMDA receptor function and affords delayed protection against ammonia toxicity and glutamate neurotoxicity. J Neuropathol Exp Neurol 54(5): 644–650.

Mirochnik VV, Ugriumov MV, Bosler O, Calas A. 2003. The effect of serotonin on differentiation of neurons producing vasoactive intestinal polypeptide in the suprachiasmatic nucleus of the rat. Neurosci Behav Physiol 33(7):729–733.

Mons N, Guillo JL, Jaffard R. 1999. The role Ca2+/calmodulin-stimulable adenylyl cyclases as molecular coincidence detectors in memory formation. Cell Mol Life Sci 55(4):525–533.

Moran J, Rivera-Gaxiola M. 1992. Effect of potassium and *N*-methyl-*D*-aspartate on the aspartate aminotransferase activity in cultured cerebellar granule cells. J Neurosci Res 33(2):239–247.

Moroo I, Tatsuno I, Uchida D, Tanaka T, Saito J, Saito Y, Hirai A. 1998. Pituitary adenylate cyclase activating polypeptide (PACAP) stimulates mitogen-activated protein kinase (MAPK) in cultured rat astrocytes. Brain Res 795(1/2):191–196.

Mycko MP, Cwiklinska H, Szymanski J, Szymanski B, Kudla G, Kilianek L, Odyniec A, Brosnan CF, Selmaj KW. 2004. Inducible heat shock protein promotes myelin autoantigen presentation by the HLA class II. J Immunol 172(1):202–213.

Mykhailyk IV, Ostapchenko LI, Kucherenko MIe. 2003. Interferon-induced 2′,5′-oligoadenylate system: Key components and biological functions. Ukr Biokhim Zh 75(3):11–21.

Nakata M, Shioda S, Oka Y, Maruyama I, Yada T. 1999. Insulinotropin PACAP potentiates insulin-stimulated glucose uptake in 3T3 L1 cells. Peptides 20(8):943–948.

Nijs J, De Meirleir K. 2005. Impairments of the 2-5A synthetase/RNase L pathway in chronic fatigue syndrome. *In Vivo* 19(6):1013–1021.

Norrholm SD, Das M, Legradi G. 2005. Behavioural effects of local microinfusion of pituitary adenylate cyclase activating polypeptide (PACAP) into the paraventricular nucleus of the hypothalamus (PVN). Regul Pept 128(1):33–41.

Nowak JZ, Kuba K. 2002. Pituitary adenylate cyclase-activating polypeptide and vasoactive intestinal peptide stimulated cyclic AMP synthesis in rat cerebral cortex slices: Interaction with noradrenaline, adrenaline, and forskolin. J Mol Neurosci 18(1/2):47–52.

Nowak LK, Zawilska JB. 1999. Adenylyl cyclase—isoforms, regulations and function. Postepy Hig Med Dosw 53(2):147–172.

Oglesbee MJ, Pratt M, Carsillo T. 2002. Role for heat shock proteins in the immune response to measles virus infection. Viral Immunol 15(3):399–416.

Onoue S, Endo K, Yajima T, Kashimoto K. 2002. Pituitary adenylate cyclase-activating polypeptide and vasoactive intestinal peptide attenuate glutamate-induced nNOS activation and cytotoxicity. Regul Pept 107(1/3):43–47.

Otto C, Zuschratter W, Gass P, Schutz G. 1999. Presynaptic localisation of the PACAP-type1-receptor in hippocampal and mossy fibres. Brain Res Mol Brain Res 66(1/2):163–174.

Pall ML. 2002. NMDA sensitisation and stimulation by peroxynitrite, nitric oxide and organic solvents as the mechanism of chemical sensitivity in multiple chemical sensitivity. FASEB J 16(11):1407–1417.

Pang RT, Lee LT, NG SS, Yung WH, Chow BK. 2004. CpG methylation and transcription factors Sp1 and Sp3 regulate the expression of the human secretin receptor gene. Mol Endocrinol 18(2):471–483.

Parsons RL, Locknar SA, Young BA, Hoard JL, Hoover DB. 2005. Presence and co-localisation of vasoactive intestinal polypeptide with neuronal nitric oxide synthase in cells and nerve fibres within guinea pig intrinsic cardiac ganglia and cardiac tissue. Cell Tissue Res. 1–13, [Epub ahead of print].

Pei L, Melmed S. 1995. Characterisation of the rat vasoactive intestinal polypeptide receptor gene 5′ region. Biochem J 308(Pt3):719–723.

Peterson PK, Molitor TW, Chao CC. 1998. The opioid-cytokine connection. J Neuroimmunol 83(1/2):63–69.

Pockley AG. 2003. Heat shock proteins as regulators of the immune response. Lancet 362(9382):469–476.

Pogribny I, Raiche J, Slovack M, Kovalchuk O. 2004. Dose-dependence, sex-and tissues-specificity, and persistence of radiation-induced genomic DNA methylation changes. Biochem Biophys Res Commun 320(4):1253–1261.

Purcell AW, Todd A, Kinoshita G, Lynch TA, Keech CL, Gething MJ, Gordon TP. 2003. Association of stress proteins with autoantigens: A possible mechanism for triggering autoimmunity? Clin Exp Immunol 132(2):193–200.

Rabl K, Reglodi D, Banvolgyi T, Somogyvari-Vigh A, Lengvari I, Gabriel R, Arimura A. 2002. PACAP inhibits anoxia-induced changes in physiological responses in horizontal cells in the turtle retina. Regul Pept 109(1/3):71–74.

Radosavljevic T, Todorovic V, Sikic B. 2004. Insulin secretion: Mechanisms of regulation. Med Pregl 57(5/6):249–253.

Rangon CM, Goursaud S, Medja F, Lelievre V, Mounien L, Husson I, Brabet P, Jegou S, Janet T, Gressens P. 2005. VPAC2 receptors mediate VIP-induced neuroprotection against neonatal excitotoxic brain lesions in mice. J Pharmacol Exp Ther 4(2):745–752.

Reina S, Sterin-Borda L, Orman B, Borda E. 2004. Human mAChR antibodies from Sjogren syndrome sera increase cerebral nitric oxide synthase activity and nitric oxide synthase mRNA level. Clin Immunol 113(2):193–202.

Runcie MJ, Ulman LG, Potter EK. 1995. Effects of adenylate cyclase-activating polypeptide on cardiovascular and respiratory responses in anaesthetised dogs. Regul Pept 60(2/3):193–200.

Samborski W, Lezanska-Szpera M, Rybakowski JK. 2004. Effects of antidepressant mirtazapine on fibromyalgia symptoms. Rocz Akad Med Bialymst 49:265–269.

Sandvik AK, Cui G, Bakke I, Munkvold B, Waldum HL. 2001. PACAP stimulates gastric acid secretion in the rat by inducing histamine release. Am J Physiol Gastrointest Liver Physiol 281(4):G997–G1003.

Schluter K, Figiel M, Rozyczka J, Engele J. 2002. CNS region-specific regulation of glial glutamate transporter expression. Eur J Neurosci 16(5):836–842.

Scott LV, Svec F, Dinan T. 2000. A preliminary study of dehydroepiandrosterone response to low-dose ACTH in chronic fatigue syndrome and in healthy subjects. Psychiatry Res 97(1):21–28.

Sethi V, Onyuksel H, Rubinstein I. 2005. Liposomal vasoactive intestinal peptide. Methods Enzymol 391:377–395.

Shaked I, Tchoresh D, Gersner R, Meiri G, Mordechai S, Xiao X, Hart RP, Schwartz M. 2005. Protective autoimmunity: Interferon-gamma enables microglia to remove glutamate without evoking inflammatory mediators. J Neurochem 92(5): 997–1009.

Sherwood NM, Krueckl SL, McRory JE. 2000. The origin and function of the pituitary adenylate cyclase-activating polypeptide (PACAP)/glucagon superfamily. Endocr Rev 21(6):619–670.

Shintani N, Suetake S, Hashimoto H, Koga K, Kasai A, Kawaguchi C, Morita Y, Hirose M, Sakai Y, Tomimoto S, Matsuda T, Baba A. 2005. Neuroprotective action of endogenous PACAP in cultured rat cortical neurons. Regul Pept 126(1/2):123–128.

Shintani N, Tomimoto S, Hashimoto H, Kawaguchi C, Baba A. 2003. Functional roles of the neuropeptide PACAP in brain and pancreas. Life Sci 74(2/3):337–343.

Shioda S, Ozawa H, Dohi K, Mizushima H, Matsumoto K, Nakajo S, Takaki A, Zhou CJ, Nakai Y, Arimura A. 1998. PACAP protects hippocampal neurons against apoptosis: Involvement of JNK/SAPK signalling pathway. Ann NY Acad Sci 865:100–110.

Shiota K. 2004. DNA methylation profiles of CpG islands for cellular differentiation and development in mammals. Cytogenet Genome Res 105(2-4):325–334.

Sommer N, Loschmann PA, Northoff GH, Weller M, Steinbrecher A, Steinbach JP, Lichtenfels R, Meyermann R, Riethmuller A, Fontana A. 1995. The antidepressant rolipram suppresses cytokine production and prevents autoimmune encephalomyelitis. Nat Med 1(3):244–248.

Sparks DL, Hunsaker 3rd, JC. 2002. Neuropathology of sudden infant death (syndrome): Literature review and evidence of a probable apoptotic degenerative cause. Childs Nerv Syst 18(11):568–592.

Srivistava P. 2002. Interaction of heat shock proteins with peptides and antigen presenting cells: Chaperoning of the innate and adaptive immune responses. Annu Rev Immunol 20:395–425.

Staines DR. 2004a. Is chronic fatigue syndrome an autoimmune disorder of endogenous neuropeptides, exogenous infection and molecular mimicry? Med Hypotheses (62):646–652.

Staines DR. 2004b. Is sudden infant death syndrome (SIDS) an autoimmune disorder of endogenous vasoactive neuropeptides? Med Hypotheses (62):653–657.

Staines DR. 2004c. Overview of an autoimmune theory of endogenous vasoactive neuropeptides. Med Hypotheses 62(5):643–645.

Staines DR. 2005a. Does dysregulation of key epigenetic and biochemical pathways occur in postulated vasoactive neuropeptide autoimmune disorders? Med Hypotheses (65):1154–1160.

Staines DR. 2005b. Do vasoactive neuropeptide autoimmune disorders explain pyridostigmine's association with Gulf War syndrome? Med Hypotheses 65:591–594.

Staines DR. 2005c. Therapeutic and preventive interventions for postulated vasoactive neuropeptide autoimmune fatigue-related disorders. Med Hypotheses (65):797–803.

Staines DR. 2005d. Do vasoactive neuropeptides and heat shock proteins mediate fatigue-related autoimmune disorders? Med Hypotheses (64):539–542.

Staines DR. 2005e. Do cytosine guanine dinucleotide (CpG) fragments induce vasoactive neuropeptide mediated fatigue-related autoimmune disorders? Med Hypotheses (65):370–373.

Staines DR. 2005f. Are vasoactive neuropeptide autoimmune fatigue-related disorders mediated via G protein-coupled receptors? Med Hypotheses (65):29–31.

Staines DR. 2006. Phosphodiesterase inhibitors may be indicated in the treatment of postulated vasoactive neuropeptide autoimmune fatigue-related disorders. Med Hypotheses 66(1): 203–204.

Stephenson TJ, Variend S. 1987. Visceral brown fat necrosis in postperinatal mortality. J Clin Pathol 40(8):896–900.

Suk K, Park JH, Lee WH. 2004. Neuropeptide PACAP inhibits hypoxic activation of brain microglia: A protective mechanism against neurotoxicity in ischemia. Brain Res 1026(1):151–156.

Tanaka S, Kuratsune H, Hidaka Y, Hakariya Y, Tatsumi KI, Takano T, Kanakura Y, Amino N. 2003a. Autoantibodies against muscarinic cholinergic receptor in chronic fatigue syndrome. Int J Mol Med 12(2):225–230.

Tanaka S, Matsunaga H, Kimura M, Tatsumi K, Hidaka Y, Takano T, Uema T, Takeda M, Amino N. 2003b. Autoantibodies against four kinds of neurotransmitter receptors in psychiatric disorders. J Neuroimmunol 141(1/2):155–164.

Tsuchiya H, Matsuda T, Harashima H, Kamiya H. 2005. Cytokine induction by a bacterial DNA-specific modified base. Biochem Biophys Res Commun 326(4):777–781.

van Eden W, Koets A, van Kooten P, Prakken B, van der Zee R. 2003. Immunopotentiating heat shock proteins: Negotiators between innate danger and control of autoimmunity. Vaccine 21(9/10):897–901.

Vaudry D, Falluel-Morel A, Basille M, Pamantung TF, Fontaine M, Fournier A, Vaudry H, Gonzalez BJ. 2003. Pituitary adenylate cyclase activating polypeptide prevents C2-ceramide-induced apoptosis of cerebellar granule cells. J Neurosci Res 72(3): 303–316.

Vaudry D, Gonzalez BJ, Basille M, Yon L, Fournier A, Vaudry H. 2000. Pituitary adenylate cyclase-activating polypeptide and its receptors: From structure to functions. Pharm Rev 52(2): 269–324.

Vaudry D, Rouselle C, Basille M, Falluel-Morel A, Pamantung TF, Fontaine M, Fournier A, Vaudry H, Gonzalez BJ. 2002. Pituitary adenylate cyclase-activating polypeptide protects rat cerebellar granule neurons against ethanol-induced apoptotic cell death. Proc Natl Acad Sci USA 99(9):6398–6403.

Waterman SA. 2001. Autonomic dysfunction in Lambert-Eaton syndrome. Clin Auton Res 11:145–154.

Waters KA, Meehan B, Huang JQ, Gravel RA, Michaud J, Cote A. 1999. Neuronal apoptosis in sudden infant death syndrome. Pediatr Res 45(2):166–172.

Watkins CC, Boehning D, Kaplin AI, Rao M, Ferris CD, Snyder SH. 2004. Carbon monoxide mediates vasoactive intestinal polypeptide associated nonadrenergic noncholinergic neurotransmission. Proc Natl Acad Sci 101(8):2631–2635.

Wei Y, Mojsov S. 1996. Tissue specific expression of different human receptor types for pituitary adenylate cyclase activating polypeptide and vasoactive intestinal polypeptide: Implications for their role in human physiology. J Neuroendocrinol 8(11): 811–817.

Whalen EJ, Travis MD, Johnson AK, Lewis SJ. 1999. Rapid tachyphylaxis to hemodynamic effects of PACAP–27 after inhibition of nitric oxide synthesis. Am J Physiol Heart Citc Physiol 276(6):H2117–H2126.

Wilderman MJ, Armstead WM. 1997. Role of PACAP in the relationship between cAMP and opioids in hypoxia-induced pial artery vasodilation. Am J Physiol 272(3Pt2):H1350–H1358.

Xu H, Strassmann G, Chan CC, Rizzo LV, Silver PB, Wiggert B, Caspi RR. 1999. Protective effect of the type IV phosphodiesterase inhibitor rolipram in EAU: Protection is independent of IL-10- inducing activity. Invest Ophthalmol Vis Sci 40(5): 942–950.

Yaka R, He DY, Phamluong K, Ron D. 2003. Pituitary adenylate cyclase activating polypeptide (PACAP(1-38)) enhances *N*-methyl-D-aspartate receptor function and brain derived neurotrophic factor expression via RACK1. J Biol Chem 278(11): 9360–9368.

Yi AK, Peckham DW, Ashman RF, Krieg AM. 1999. CpG DNA rescues B cells from apoptosis by activating NFkappaB and preventing mitochondrial membrane potential disruption via a chloroquine sensitive pathway. Int Immunol 11(12): 2015–2024.

Zawilska JB, Dejda A, Niewiadomski P, Gozes I, Nowak JZ. 2005. Receptors for VIP and PACAP in guinea pig cerebral cortex: Effects on cyclic AMP synthesis and characterization by 1251-VIP binding. J Mol Neurosci 25(3):215–224.

Zhang Z, Weinschenk T, Schluesener HJ. 2005. Uptake, intracellular distribution, and novel binding proteins of immunostimulatory CpG oligodeoxynucleotides in microglial cells. J Neuroimmunol 160(1/2):32–40.

Zhou CJ, Shioda S, Yada T, Inagaki N, Pleasure SJ, Kikuyama S. 2002. PACAP and its receptors exert pleiotropic effects in the nervous system by activating multiple signalling pathways. Curr Prot Pep Sci (3):423–439.

